# On the Reliability of HZO-Based Ferroelectric Capacitors: The Cases of Ru and TiN Electrodes

**DOI:** 10.3390/nano12173059

**Published:** 2022-09-03

**Authors:** Roman R. Khakimov, Anna G. Chernikova, Aleksandra A. Koroleva, Andrey M. Markeev

**Affiliations:** Moscow Institute of Physics and Technology, Institutskii per. 9, 141700 Dolgoprudny, Russia

**Keywords:** ferroelectricity, hafnium oxide, Hf_0.5_Zr_0.5_O_2_, ruthenium, reliability, fatigue, endurance, wake-up, retention, imprint

## Abstract

Despite the great potential of Hf_0.5_Zr_0.5_O_2_ (HZO) ferroelectrics, reliability issues, such as wake-up, fatigue, endurance limitations, imprint and retention loss, impede the implementation of HZO to nonvolatile memory devices. Herein, a study of the reliability properties in HZO-based stacks with the conventional TiN top electrode and Ru electrode, which is considered a promising alternative to TiN, is performed. An attempt to distinguish the mechanisms underlying the wake-up, fatigue and retention loss in both kinds of stacks is undertaken. Overall, both stacks show pronounced wake-up and retention loss. Moreover, the fatigue and retention loss were found to be worsened by Ru implementation. The huge fatigue was suggested to be because Ru does not protect HZO against oxygen vacancies generation during prolonged cycling. The vacancies generated in the presence of Ru are most likely deeper traps, as compared to the traps formed at the interface with the TiN electrode. Implementing the new procedure, which can separate the depolarization-caused retention loss from the imprint-caused one, reveal a rise in the depolarization contribution with Ru implementation, accompanied by the maintenance of similarly high imprint, as in the case with the TiN electrode. Results show that the mechanisms behind the reliability issues in HZO-based capacitors are very electrode dependent and simple approaches to replacing the TiN electrode with the one providing, for example, just higher remnant polarization or lower leakages, become irrelevant on closer examination.

## 1. Introduction

Since the first discovery of ferroelectricity (FE) in HfO_2_-based thin films, many experimental works showed the great potential of these materials for the development of ferroelectric random access memory (FRAM) [1]. In particular, Hf_0.5_Zr_0.5_O_2_ (HZO) mixed oxide is considered to be especially promising for the most traditional concept of FRAM with a control transistor and metal-FE-metal (MFM) capacitor because it can be crystallized to the FE orthorhombic phase at 400 °C, compatible with back-end-of-line (BEOL) process flow [1,2,3]. In addition, HfO_2_ (HZO)-based capacitors are more promising compared to capacitors with traditional FEs (Pb(Zr, Ti)O_3_ (PZT), BaTiO_3_, etc.) because of the compatibility with complementary metal-oxide-semiconductor (CMOS) technology, low physical thickness, etc. However, they show similar performance instability and reliability issues, which make the advantages meaningless and hamper FRAM development. Among these issues are: (*I*) wake-up effect or rise of the switching polarization with cycling, which is observed at the initial cycling stage, (*II*) fatigue effect, which is the cycle-by-cycle decrease in the remnant polarization value observed after a certain number of cycles, (*III*) endurance limitations, (*IV*) imprint or the gradual shift of the hysteresis curve toward positive/negative electric field when upward (P↑)/downward (P↓) polarization state is stored, which is one of the major contributors to the (*V*) retention loss phenomenon.

To date, many works have evaluated the physical mechanisms behind these phenomena. The wake-up effect is usually attributed to the oxygen vacancies (V_Os_) and O^2−^ ion re-distribution under an electric field, followed by creating a more uniform field within HZO, which leads to the involvement of new domains to the polarization switching directly and/or indirectly (through the phase transformation from the non-ferroelectric to the ferroelectric phase) [4]. Alternatively, recently, it was shown that ferroelastic switching may explain the wake-up in HZO-based capacitors [5].

Two main mechanisms proposed previously to explain fatigue in PZT-based capacitors, i.e., domain wall pinning (DWP) and seed inhibition [6], are considered to explain fatigue in HfO_2_-based capacitors. Huang et al. [7] suggested that DWP is the most relevant mechanism for capacitors based on Y-doped HfO_2_. DWP is attributed to the interactions between the mobile charges and bound charges in the domain walls. Noteworthily, it was reported that V_Os_ are hardly the reason for DWP because of the higher V_O_ generation energy as compared to the observed fatigue activation energy. On the contrary, Pešić et al. [4] attributed fatigue to V_O_ generation in Sr:HfO_2_ at the FE/electrode interfacial layer, followed by the electrons trapping at V_Os_, which affects the field distribution within the stack, reducing the field in the bulk of the ferroelectric material. Similarly, in a report by Mittmann et.al. [8], where, unlike previously mentioned works, TiN electrodes were replaced by IrO_2_, the formation of an interfacial layer and reduction in the electric field in the bulk of HZO is also suggested to be the origin of fatigue. However, Huang et al. showed the recovery of the fatigued device polarization by mild heating, which, according to the authors, excludes the formation of an additional field screening layer and suggests that FE/electrode interfaces play a very minor role in fatigue [7]. Thus, there is no agreement on the origin of fatigue in HfO_2_-based capacitors. On the other hand, better certainty has been achieved in the origin of endurance limitation. It is attributed to the generation of V_Os_ during cycling, an increase in the leakages, Joule heating and consequent hard breakdown of FE [4,9,10].

Considering imprint and related retention loss phenomenon (the former seems to be one of the major contributors to the last one [11,12]), in PZT-based capacitors, they are usually ascribed under the interfacial screening model (ISM) [13]. According to ISM, the presence of a non-FE interfacial layer (IL) at the FE/electrode interface leads to the spatial separation of the bounded polarization charges in the FE layer and free screening charges in metal electrodes. As a result, a strong electric field across IL arises, leading to charge injection from the electrode to the FE layer, trapped by V_Os_ and the appearance of the built-in electric field (imprint). Despite the lack of exhaustive research, ISM can be applied to HfO_2_-based capacitors too [14,15].

As is evident from this discussion, overall defects in the ferroelectric material (presumably, V_Os_) play an essential role in the reliability of HfO_2_-based stacks. It is well accepted also that different electrode materials and processing conditions lead to the different V_O_ concentrations in the FE layer because of the variation in the thermally stimulated oxygen-scavenging ability of the electrodes. It leads, on the one hand, to the different phase composition and polarization values, because V_O_ concentration affects the relative stability of ferroelectric and non-ferroelectric phases [16,17]. On the other hand, reliability issues also cannot be independent of the electrode materials and processing conditions.

In this regard, any attempt to replace conventional TiN should start with a comprehensive analysis of the resultant reliability. Recently, special attention was paid to Ru electrodes [18,19]. An interest in Ru is explained mostly by improved chemical stability, potentially more stable interface with HfO_2_, high work function and the existence of the dry plasma etching processes, selective to HfO_2_. The excellent ferroelectric response and improved endurance were shown for HZO-based capacitors with Ru top and bottom electrodes grown by magnetron sputtering [18]. In turn, according to our previous work [19], the bottom Ru electrode provides a more preferred texture of o-phase grains in HZO and a high polarization value. However, a more rapid breakdown instead of endurance improvement was observed [19]. In addition, careful analysis of both papers reveals more prominent fatigue of stacks with Ru electrodes in comparison to TiN [18,19]. To the best of our knowledge, the other reliability issues, such as imprint or wake-up, have not been investigated to date. Overall, there remains huge uncertainty in the influence of Ru on reliability issues in HZO-based capacitors.

Within this study, we examine the influence of the Ru electrode on the reliability of the fully ALD-grown HZO-based stacks. To provide more value in terms of future technological applications, in this work, we specifically chose full ALD growth with a low thermal budget (400 °C) and *automatic annealing* conditions (will be explained below) because such a processing flow potentially meets the BEOL requirements.

## 2. Materials and Methods

Within this work, two types of structures with different top electrodes were formed and investigated: TiN/HZO/TiN and TiN/HZO/Ru/TiN referred to as HZO capacitors with TiN and Ru TE below, respectively. Thus, 3-inch Si (10 Ω·cm) wafers with 100 nm thick plasma-enhanced chemical vapor deposited SiO_2_ (for insulation) and 20 nm thick sputtered W layer (for reduction of BE resistance) were used as substrates. HZO, TiN and Ru TE were grown by ALD in the R200adv (Picosun) tool. Further, 10 nm thick HZO was grown from tetrakis(ethyl-methyl-amino) hafnium (TEMAH), tetrakis(ethyl-methyl-amino) zirconium (TEMAZ) and H_2_O at a deposition temperature (T_dep_) of 240 °C. Pulse/purge times of TEMAH, TEMAZ and H_2_O were 0.5 s/12 s, 0.5 s/12 s and 0.1 s/12 s, respectively. The ratio of alternate TEMAH-H_2_O and TEMAZ-H_2_O cycles was 1:1 and the total number of supercycles needed for the growth of ~10 nm thick HZO was 65. The Hf:Zr atomic ratio was ≈0.8 according to X-ray photoelectron spectroscopy (spectra are not shown) which is relevant to the Hf_0.45_Zr_0.55_O_2_ composition of HZO. Next, for the Ru TE case, 3 nm thick Ru was grown on HZO by radical-enhanced ALD (REALD) at a T_dep_ of 300 °C using bis(ethyl-cyclopentadienyl) ruthenium (Ru(EtCp)_2_) and oxygen radicals (O*), as described previously [20]. TiN (10 nm thick for BE and 20 nm thick for TE) was grown by thermal ALD at a T_dep_ = 400 °C using TiCl_4_ and NH_3_. ALD process for TiN TE served also for crystallization of the underlying HZO (*automatic annealing*). This process takes ≈4 h. No additional RTA was applied. 

The structural properties of all HZO films after the *automatic annealing* were examined by grazing-incidence X-ray diffraction (GIXRD) on the ARL X’TRA (Thermo Fisher Scientific, Waltham MA, USA) tool with Cu Kα radiation using an incident angle of 1°. The spectra were collected with a 0.02° step resolution and integration time of 40 s, with the presence of TE.

For electrical measurements, top contact pads with an area of ≈2 × 10^−5^ cm^2^ were formed in the TE using photolithography, followed by plasma etching of TiN and Ru in SF_6_/Ar and O_2_/Ar, respectively. Further, 50 nm thick Al layer was grown both on top and bottom contact pads to improve contact resistance and stability. Small-signal capacitance-voltage (C-V) measurements with an AC signal frequency of 10 kHz and amplitude of 50 mV were used to estimate the dielectric constant (*k*) through the parallel-plate capacitor formula. Quasi-static switching current (I_sw_)-electric field (E) curves were measured in response to a triangle waveform with a frequency of 0.4 kHz and voltage amplitude of 3 V. Retention data were measured using trapezoidal voltage pulses with a duration of 3 μs (1 μs rise and 1 μs fall times) and an amplitude of 3 V. Dynamic I_sw_-E curves were measured in response to the triangle waveform with the frequency corresponding to the rate of voltage increase in pulse measurements, i.e., 250 kHz. Polarization hysteresis loop reconstruction was derived by integration of the I_sw_. Endurance measurements were performed using bipolar trapezoidal voltage pulses with different amplitudes and a constant pulse duration of 3 μs. Positive-up-negative-down (PUND) method was used to measure I_sw_-E, polarization hysteresis loops and endurance. All electrical measurements were performed on a Summit 11000B-M (Cascade Microtech, Beaverton, OR, USA) probe station by the Agilent B1500A (Agilent Technologies, Santa Clara, CA, USA) semiconductor analyzer.

## 3. Results and Discussion

### 3.1. Crystalline Structure

**Figure 1:**
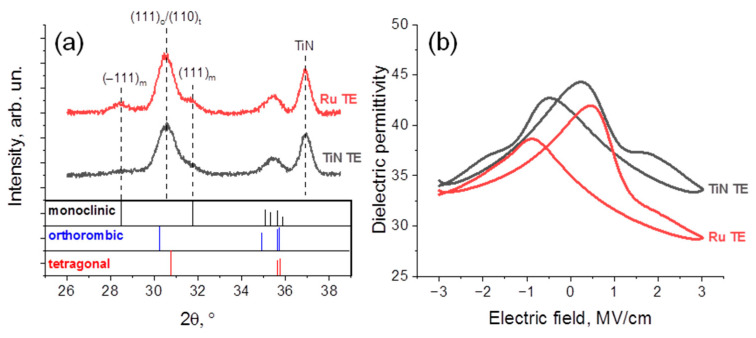
(**a**) GIXRD for HZO-based capacitors with TiN and Ru TE with reference patterns reported for m-, o- and t-phases of HZO [1]. (**b**) *k* vs. applied electric field dependences obtained from the small-signal C-V measurements performed at the pristine state for HZO-based capacitors with TiN and Ru TE.

First, we analyzed the crystalline structure of HZO with TiN and Ru TE. According to GIXRD data presented in Figure 1a within a 2θ range 26–38.5°, where the most intensive peaks from HZO are observed, in all cases, *automatic annealing* resulted in HZO crystallization to a combination of non-FE t-phase (space group *P4_2_/nmc*) and m-phase (space group *P2_1_/c*) and FE o-phase (space group *Pca2_1_*). Overall diffraction patterns look very similar, except for the deviation between m-phase fractions that may be concluded safely. Calculated as I_m_/(I_o/t_ + I_m_) within a 2θ range 25–33°, where I_m_ is net intensity of m(−111) and m(111) reflections, I_o/t_ is a net intensity of (111_)o_/(110_)t_ reflections; the m-phase relative intensity increased from ≈22% to ≈38% when TiN TE was replaced by Ru TE. Although the precise deconvolution of the peaks responsible for the t- and o-phases cannot be performed because of the structural similarities, indirect approaches may propose their relative concentrations [2]. Typically, the analysis of the *k* value far from the switching maximums derived from the small-signal C-V curves is performed, because three phases commonly observed in HZO have very different *k*-values: 16–20, ~30 and 35–40 for m-, o- and t-phases, respectively [2], so they affect the *k* value measuring macroscopically.

Note that C-V curves obtained at the pristine states of capacitors (Figure 1b) are essentially asymmetric and *k*-values are different at voltages −3 V and at 3 V, both far from the switching maximums. Because the shape of the small-signal C-V curve can be influenced not only by the ferroelectric polarization but also by the polarization of domain walls, which can have different configurations at the opposite polarization states, we believe it is fairer to compare the smallest *k*, i.e., at 3 V in our case. According to Figure 1b, *k* ≈ 35 and ≈29 at the voltage of 3 V. Lower *k* of HZO measured from a device with Ru TE correlates with a higher m-phase fraction possessing the lowest *k*-value. However, the relatively high *k* of HZO with TiN TE allows us to propose that HZO with TiN TE comprises a slightly higher portion of the highest-*k* non-FE t-phase. It should be noted that despite the above-discussed asymmetry, in principle, the higher *k* of HZO with TiN TE is also observed for the voltage of −3 V, which implies consistency in our suggestion. The increase in the m-phase fraction in the case of the Ru TE is most likely related to the elimination of the scavenging effect from the top TiN electrode since, previously, it was shown that the oxygen content increase tends to stabilize the m-phase in HZO films [16]. Alternatively, the preparation procedure for TE may also affect the observed differences. Namely, in the case of Ru TE, the surface of HZO underwent the O* treatment at the first stages of Ru growth by the Ru(EtCp)_2_/O* REALD process, which may also contribute to the increasing content of oxygen. In addition, one cannot exclude the crystalline structure of HZO in the devices with TiN and Ru TE may be sensitive to conditions at which the annealing and, specifically, its cooling stage occur. Because T_dep_ of Ru equaling 300 °C is not enough for the HZO crystallization [21], HZO layers in both cases are expected to crystallize during an identical automatic annealing procedure. Therefore, the cooling stage occurs in the presence of the top TiN and Ru/TiN bilayer in the case of TiN and Ru TE, respectively. Different mechanical confinement may, in principle, lead to variation in the crystalline structure.

### 3.2. Cycling Performance

**Figure 2:**
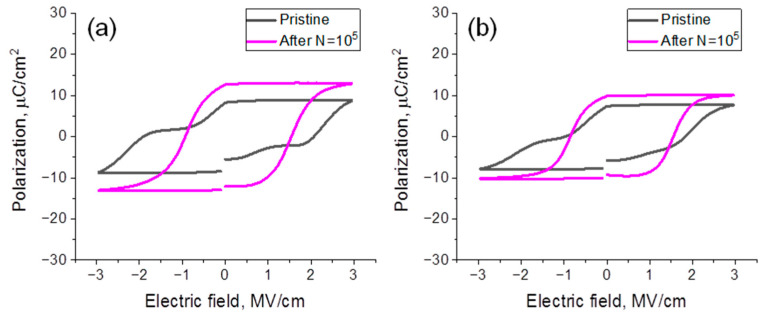
Polarization hysteresis loops measured from the devices with TiN (**a**) and Ru (**b**) TE in pristine and woken-up states.

In Figure 2, P-E hysteresis measured quasi-statically at the pristine state and after the wake-up procedure (application of 10^5^ switching cycles (N) using bipolar voltage pulses with 3 µs duration and 3 V amplitude) from the capacitors with TiN and Ru TE is depicted. In the pristine state, the double remnant polarization (2P_r_) of capacitors with TiN and Ru TE was ≈18 µC/cm^2^ and ≈16 µC/cm^2^, respectively. In the woken-up state, 2P_r_ became higher, ≈26 µC/cm^2^ and ≈19 µC/cm^2^, respectively. The higher 2P_r_ value and more prominent wake-up (30% vs. 24% of the final 2P_r_ value) of the capacitor with TiN TE resemble well the above-discussed structural changes. In particular, higher m-phase content in HZO with Ru TE is suggested to account for the lower 2P_r_. At the same time, a slightly higher portion of t-phase in the film with TiN TE may be responsible both for a more pinched hysteresis loop in the pristine state (black lines in Figure 2) and more pronounced wake-up, because it is suggested to transform to FE o-phase during wake-up easier than the m-phase due to the lower energy difference [22].

**Figure 3:**
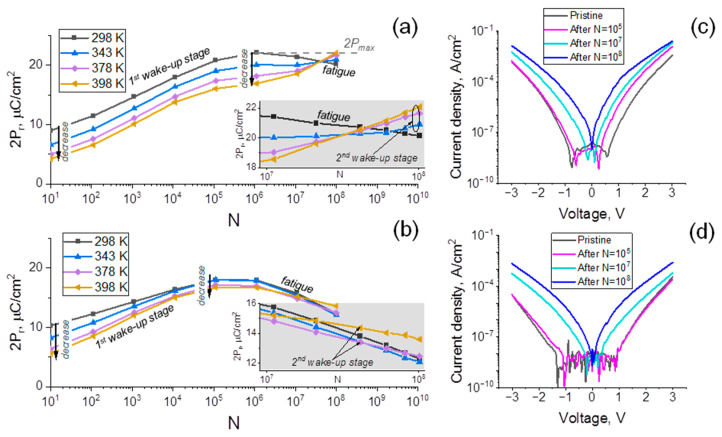
2P_r_ as a function of the number of switching cycles measured from devices with TiN (**a**) and Ru (**b**) TE at T_amb_ ranging from 298 K (RT) to 398 K. Insets: enlarged 2P_r_ (N) dependences at the N ranging from 10^7^ to 10^8^. The measurements were performed using 3 µs, 3 MV/cm pulses. (**c**,**d**) Leakage current densities measured from devices with TiN (**c**) and Ru (**d**) TE at RT.

Figure 3a,b demonstrate the result of cycling tests performed at different ambient temperatures (T_amb_), from which the quantitative differences in wake-up may also be confirmed. One can observe the prominent wake-up in the region between N = 10 and N = 10^5^–10^6^ for both devices and 2P_r_ raises on 58% for the device with TiN TE and only on 37% for the one with Ru TE, respectively, at room temperature (RT) conditions. Note that qualitatively higher wake-ups in both cases as compared to Figure 2 arise from the difference between the waveforms applied for the measurements. It is expected that lower-frequency-probing wakes up the device by itself, leading to the higher 2P_r_ in a pristine state.

It is also helpful to consider the results obtained at elevated T_amb_ to investigate mechanisms responsible for the cycling behaviors, including wake-up. Initially measured 2P_r_ (in Figure 3a,b measurements start from N = 10) tends to decrease with the rise in T_amb_ (in a range 298–398 K) for both devices. Although maximum 2P_r_ (measured at N~10^5^–10^6^) decreases rather notably with the rise of the T_amb_ for the device with TiN TE and not so dramatic for the device with Ru TE, there is a general tendency for a decrease in the maximum 2P_r_ value too.

At elevated T_amb_, rather complex cycling behavior becomes obvious in both cases. In the device with TiN TE, the slope of 2P_r_ (N) dependence at high N (10^7^–10^8^) reverses its sign from a negative value (which indicates fatigue) to a strongly positive one already at 343 K, which means the activation of a concurrent wake-up process at elevated T_amb_ (inset to Figure 3a). This newly activated wake-up will be denoted as the second stage of wake-up below, while the whole wake-up process will be denoted as a *two-step wake-up*. The slope of 2P_r_ (N) dependence rises with a further increase in T_amb_ and finally almost approaches T_amb_ = 398 K, the maximum 2P_r_ achieved at RT after N~10^5^–10^6^ (2P_max_ marked with a dotted line in Figure 3a).

In the device with Ru TE, the 2P_r_ (N) slope at high N remains negative in all T_amb_ ranges; however, its absolute value decreases slightly at T_amb_ = 378 K as compared to lower T_amb_. Such a decrease becomes more noticeable at T_amb_ = 398 K. This also means the activation of the second stage of wake-up at elevated T_amb_. However, the struggle between two competing processes, fatigue and the second stage of wake-up finishes in favor of the former because the fatigue effect is very strong in the case of Ru TE.

Thus, *two-step wake-up* is the common property in both devices at elevated T_amb_. Although fatigue is observed also at RT for both devices, it is more prominent for Ru TE implementation; thus, despite the second stage of wake-up, it is conserved even at elevated T_amb_.

Turning to the analysis of mechanisms, it should be noted that the above-discussed decrease in 2P_r_ at the first wake-up stage with the rise in T_amb_ in both cases, suggests that the phase change between non-FE t-phase and FE o-phase underlies wake-up, because the non-FE t-phase becomes relatively more stable with the rise in T_amb_, resulting in lower 2P_r_ at each N [23]. According to the literature, re-distribution and/or generation of V_Os_ during cycling may account for such phase transformations. To note, the redistribution of existing V_Os_ usually leads to the depinning of individual domains, which manifests itself not only in the rise in 2P_r_ but also in the merging of polarization switching peaks and eventually de-pinching of FE hysteresis [4]. The related transformation of the FE hysteresis from the pinched to the open curve is clear for the first wake-up step (up to N = 10^5^) for both devices with TiN and Ru TE (Figure 2). At the same time, the possibility of generation of V_Os_ at this step is worth analyzing additionally. Figure 3c,d shows the leakage current densities measured from the stacks at the pristine state and after 10^5^, 10^7^ and 10^8^ switching cycles. It should be noted that the leakages in such devices may be explained by bulk or interface-limited mechanisms. Considering the bulk-type mechanisms, the increased concentration of traps is expected to directly affect the charge transfer probability. Considering the barrier-type ones, V_Os_ could affect the effective work function of the electrode, which will also influence the injection probability. It is reported that oxygen deficiency at the HfO_2_/Ru interface leads to a decrease in Ru work function [24]. Thus, independently of the exact leakage mechanism, the generation of V_Os_ is expected to result in higher leakages. According to Figure 3d, the leakage current density does not rise with Ru TE during the first 10^5^ cycles, which shows that the V_O_ generation is unlikely and the re-distribution of existing V_Os_ is more possible.

A different situation is observed in the device with TiN TE (Figure 3c). The leakages increase at positive polarity after 10^5^ cycles, showing the possibility of increasing the number of V_Os_. In principle, V_O_ generation during cycling, on the one hand, may be explained by higher chemical activity of TiN and consequent higher scavenging ability [25]. Some V_O_ generation could, in principle, explain a more prominent lowering of 2P_r_ with a rise in T_amb_ at every stage of the field cycling process, as compared to the case of Ru TE (*i*) and easier activation of the second wake-up step of the *two-step wake-up* in devices with TiN TE (*ii*). Indeed, it is known that the t-phase becomes more stable at higher temperatures and higher V_O_ content [9,26]. Thus, the excess of V_Os_ generated during cycling may change the mutual stability of the t- and o-phase and, eventually, (*i*). However, this excess of V_Os_ will also be re-distributed during the following cycling (N > 10^5^), which may cause the activation of the prominent second wake-up step at elevated T_amb_ (through t- to o-phase transformation). Noteworthily, the strongest second stage of wake-up is observed at the highest T_amb_, but even this strongest 2P_r_ rise is limited by the maximum 2P_r_ achieved at RT (2P_max_ marked with a dotted line in Figure 3a for the TiN TE case). Such a behavior, indeed, indicates that a similar process underlies the differently looking wake-ups at RT and elevated T_amb_ (the maximum phase transformation occurred during first stage of wake-up at RT, while the changed V_O_ content delayed such a transformation, leading to the second stage of wake-up occurrence at elevated T_amb_). In addition, a similar process seems to be responsible for wake-ups in the devices with different TE (t- to o-phases transformation because of the V_O_ re-distribution and generation (*first scenario*)), but it flows in slightly different conditions. With Ru TE, generation of additional V_Os_ is delayed and leakages increase at higher N (Figure 3c,d); thus, the second stage of wake-up activates late. 

It should be noted that previously, *the two-step wake-up* was already observed [23]. In that case, the authors suggested that the partial breakdown of the interfacial non-FE layer, which is always present in HZO-based capacitors, occurs during cycling. At some stage of cycling, higher voltage drops at HZO, resulting in another round of 2P_r_ increases. This stage should be closely related to reductions in the device’s conductivity. In principle, this is the alternative way to connect the increase in leakages after N = 10^5^, observed in Figure 3c at positive electric fields with a more prominent *two-step wake-up* in the device with TiN TE. The polarity at which it happens shows that breakdown should occur at the bottom interface with TiN, in particular, in TiO_x_N_y_/TiO_2-x_, always present at the bottom interface because of the air exposure and/or chemical reactions with HZO [27,28]. Although such an approach to explaining *two-step wake-up* (*second scenario*) could not be excluded, it is not very likely, because one does not expect the critical difference between the bottom interfaces of devices with TiN and Ru TE. We have to note also that, at this step, we cannot exclude the possibility that wake-up in both devices is explained by the ferroelastic switching (*third scenario*) [29]. Recently, it was shown that wake-up may be related to the transformation of ferroelectric domains from in-plane orientation, induced by the in-plane tensile strain arising in the structure during heating and cooling down, to the out-of-plane one [5,30,31]. Both devices are expected to be under relatively similar strain, induced mostly by the thick substrate, which may explain qualitatively similar wake-up. Some differences may arise from the different confinements between TEs. Additional experiments are required to verify this possibility. 

**Figure 4:**
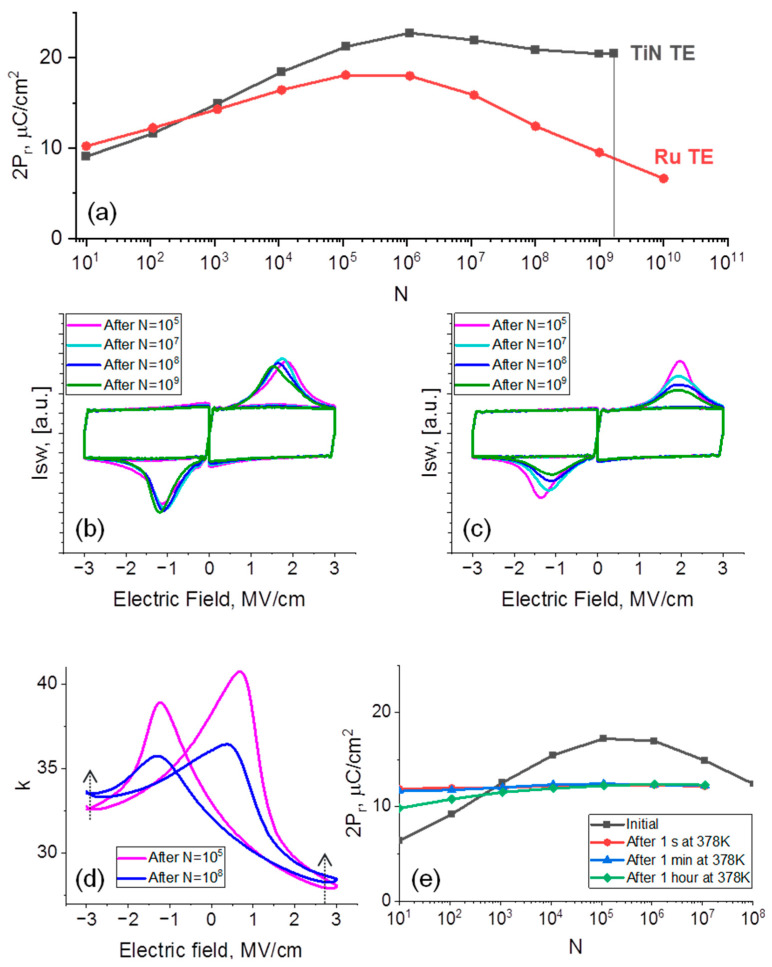
(**a**) 2P_r_ as a function of the number of switching cycles measured from devices with TiN and Ru TE at RT; dynamic I_sw_-E curves measured after N = 10^5^, 10^7^, 10^8^ and 10^9^ from devices with TiN (**b**) and Ru (**c**) TE; *k*-value dependence on E, extracted from the small-signal measurements of the device with Ru TE after N = 10^5^ and 10^8^ (**d**), inset: dependence of *k*-value at 3 MV/cm on N; (**e**) results of polarization recovery test: initially recorded endurance up to N = 10^8^ ended up with the decrease in 2P_r,_ i.e., fatigue and endurances measured after the baking of the fatigued device at 105 °C during different baking times.

The comparison of leakages in devices with Ru and TiN TE reveals also that leakages are considerably lower in all steps of cycling in devices with Ru TE, which may lead to serious consequences. First, the lower leakages at the pristine states are an indicator of lower net Vo content in the device with Ru TE (because of lower chemical activity and scavenging ability, which is also in accordance with the increase in the m-phase content discussed above), which is believed to be a reason for qualitatively less prominent wake-up in all stages because of the lower t-phase content (t-phase is expected to be more preferred at high V_O_ content). In addition, as mentioned above, the leakage current induces cumulative Joule heating during repeated cycling, leading to higher breakdown probability and endurance worsening. Figure 4a demonstrates the comparison of endurance measurement results at RT. Capacitor with TiN TE breaks down after ~1 × 10^9^ switching cycles (at 3 µs/3 V voltage pulses), while the ones with Ru TE continue to work after at least 10^10^ switching cycles.

Despite the observed endurance improvement, as mentioned above and clear from Figure 4a, dramatic fatigue is a distinctive property of the device with Ru TE. 2P_r_ drops to ≈7 µC/cm^2^ after N = 10^10^, while the fatigue of the device with TiN TE is almost negligible. As discussed in the introduction, to date, there is no clear understanding of the origin of fatigue. Microscopically, DWP, seed inhibition, phase transformation and formation of the interfacial screening layer are possible. The fourth mechanism may be verified relatively easily. If the interfacial screening layer forms somehow during cycling, then depending on the exact trapping/de-trapping energy, the permanent or recoverable electric charge will be accumulated at this layer during cycling. It means the screening electric field will occur and, consequently, the I_sw_-E curves should systematically broaden and/or shift, so that the applied electric field will not be enough for complete polarization reversal, as was observed by Mittmann et al. [8]. Figure 4b,c represent the dynamic I_sw_-E curves, measured from the devices with TiN and Ru TE, respectively. I_sw_-E neither measured from a device with TiN nor from the one with Ru becomes broader with the increase in N. Just a decrease in the I_sw_ peak amplitude is visible in the case of a device with Ru TE, which shows the “turning off” of some portion of switchable polarization, without the appearance of any built-in field or drop in the portion of applied field at the interfacial layer. This means that interfacial layer formation may likely be ruled out from the consideration in this case. However, the choice between the left three mechanisms is rather challenging. To gain insight into the topic, analysis of permittivity change may be helpful. As shown in Figure 4d, the *k* values far from the switching maximums are higher at N = 10^8^ (which is a fatigued state) than *k* at N = 10^5^ (woken-up state). In particular, it increases from 32.5 to 33.5 and from 28 to 28.5 for −3 V and 3 V, respectively. Noteworthily, the slight increase in *k* during fatigue is expected for DWP. The fatigued device caused by DWP contains more immobilized domains in the fully poled state than the poled state after N = 10^5^. As a result, the poled fatigued state would contain more domain walls. These walls will additionally contribute to the small-signal *k*, making the measured *k* value higher than the one at the poled state after N = 10^5^. However, such a change is not unique to DWP. The increase in *k* is expected also for the change transformation to the t-phase. However, the rise in the *k* value is not expected for the seed inhibition mechanism. Although the seed inhibition is then unlikely, in principle, additional investigations are required to rule it out with higher certainty mostly because of the marginal change in *k* detected in our work (Figure 4d). 

Thus, at this point, DWP and phase transformation seem more likely to contribute to fatigue. Both mechanisms in HZO-based capacitors may be attributed to the generation of additional V_Os_ with cycling, which can be easily suspected from Figure 3d due to the increase in the leakages with increasing N > 10^5^. In the case of DWP, additionally generated V_Os_ may be the domain wall-trapping defects. Noteworthily, previously low activation energy of the fatigue and recovery of fatigue after mild heating were taken as a confirmation that V_Os_ are unlikely the domain wall-trapping defects [7]. In our work, we do not see any fatigue activation with the increase in T_amb_ from RT to 343 K (compare slopes of 2P_r_ (N) at RT and T_amb_ = 343 K in the inset to Figure 3b). However, even worse, fatigue is superimposed with the second wake-up stage at T_amb_ > 343 K (Figure 3b and inset to Figure 3b). Thus, a direct measurement of activation energy of fatigue is not possible. We performed a recovery test instead. After cycling during N = 10^8^, we baked the device with Ru TE at 105 °C for 1 s, 1 min and 1 h and performed the cycling again after each of the baking times. We detected no recovery of fatigue (Figure 4e). Instead, even lower 2P_r_ (≈9 µC/cm^2^) as compared to the fatigued state (≈12 µC/cm^2^) is observed right after the longest bake (1 h), which is easily explained by imprint, occurred because of the baking at elevated temperature. This slightly lower 2P_r_ approaches the fatigued state 2P_r_ (~12 µC/cm^2^) after 10^7^ switching cycles and saturates at this value with no signs of recovery to the initial non-fatigued 2P_r_ (~18 µC/cm^2^). Thus, unlike previous reports, we achieved completely different results. This means that, in principle, deep trapped defects, including V_Os_, may account for the fatigue in the device with Ru TE. At the same time, the precise investigation of the crystal structure evolution of the device with Ru TE with continuous cycling is still required to verify what mechanism, DWP or phase transformation, or both, accounts for the fatigue. However, there is also an open question about the precise role of Ru. It is evident that Ru promotes undesired fatigue as compared to TiN. One of the explanations is the formation of deeper defects, responsible for fatigue at Ru/HZO interfaces as compared to TiN/HZO. Under the DWP mechanism, if one accepts it for both devices, with TiN and Ru TE, shallower trapped defects may be de-trapped during cycling, resulting in almost no fatigue (TiN TE case), while deeper defects would lead to persistent fatigue (Ru TE case).

The presented discrepancy between the previously reported results emphasizes that very different effects can be observed in the HZO system, depending on the electrode materials, formation and post-processing procedures. In addition to the complexity in each of the effects, they superimpose, which results in exceptional challenges for their investigation. Overall, new approaches to profound investigations are needed to get a deeper insight into the nature of the cycling performance and, exceptionally, fatigue.

### 3.3. Retention Loss

To further elucidate the effect of Ru TE on the reliability properties in HZO-based capacitors, we performed retention measurements. A variation of the procedure reported by Mueller et al. [32], which allows one to estimate the evolution of the so-called same state (SS), new same state (NSS) and opposite state (OS), was used. The baking times were 10, 100, 1000 and 5000 min and the baking temperature was 85 °C.

**Figure 5:**
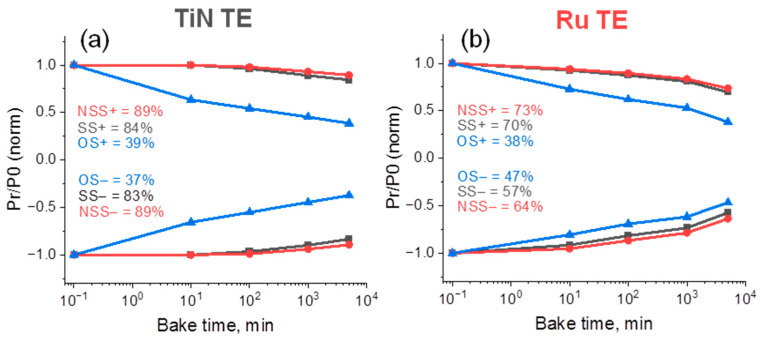
Retention at 85 °C of SS, NSS and OS states for the upward P↑ (SS^+^, NSS^+^ and OS^−^) and downward P↓ (SS^−^, NSS^−^ and OS^+^) polarization states each versus bake time for HZO-based capacitors with TiN (**a**) and Ru (**b**) TE. Measurements were performed using trapezoidal voltage pulses with a duration of 3 μs (1 μs rise and 1 μs fall times) and amplitude of 3V.

As was expected from the previous study, where RTA at 400 °C led to a rapid retention loss of all kinds of states (and especially OS) [12] in TiN/HZO/TiN capacitor, *automatic annealing* at 400 °C, the device with TiN TE also resulted in a valuable retention loss. Although moderate retention loss was observed for the SS and NSS (less than ~17% was lost after 5 × 10^3^ min at 85 °C), the retention loss of OS states exceeded 60% after 5 × 10^3^ min of baking (Figure 5a). Only a slight improvement in the retention of OS^−^ occurs when TiN TE is replaced by Ru (Figure 5b). However, the SS and NSS degraded even more severely than with TiN TE. For example, ~17% and ~43% of SS^−^ were lost after 5 × 10^3^ min of baking at 85 °C for the devices with TiN and Ru TE, respectively. Generally, retention of different types of states (SS (NSS) and OS) became mutually more similar when TiN was replaced by Ru.

The phenomena underlying the retention loss in ferroelectric capacitors are usually divided into three types: relaxation, depolarization and imprint [33,34]. Relaxation is a rapid decrease in polarization in the first seconds after the removal of the external electric field. Figure 5 shows that this phenomenon, if it exists, does not contribute valuably to the retention loss, because the retention loss problem enlarged at higher time scales. Usually, under depolarization, several types of phenomena are implied: (*i*) thermal depolarization induced by the phase transformation at temperatures approaching the Curie temperature (T_C_) and (*ii*) depolarization induced by an imperfect screening of the polarization charges and the occurrence of the depolarization field (E_dep_) because of the non-ferroelectric IL formation [35] and/or non-ferroelectric grains in the bulk [36]. It should be noted that the (*i*) type of depolarization can be excluded safely from consideration because of the above-shown prominent ferroelectric response in both devices, even at higher T_amb_ than was applied in the retention experiment. In contrast, type (*ii*) depolarization is widely discussed in the literature and applied to explain retention loss, especially when an imprint fails to explain it exhaustively [36]. At the same time, the imprint is still considered to be the major contributor to retention loss in HZO-based capacitors [11,12]. Thus, the main question for the discussion is which mechanism, depolarization, imprint or both, handles the retention loss in the devices with TiN and Ru TE.

In this regard, we have to emphasize again that these two devices show very different features of retention loss. The device with TiN TE shows a rather moderate loss of SS polarization with baking time and huge OS loss (Figure 5a). This phenomenon is irrelevant for depolarization. Indeed, it can be understood that depolarization, manifesting itself in the disorientation of some domains because of the imperfect screening of ferroelectric polarization, should affect primarily the SS state and because of the conservation of charge law, the OS state too. Eventually, very similar SS and OS retentions are expected, as pictured in Figure 5b. Instead, the highest loss in the OS state, observed for the device with TiN TE in Figure 5a, is more typical for the imprint phenomenon. In the imprint case, the difference between the SS and OS states arises because the spontaneous back switching occurs at the fall of the voltage pulse applied for the SS reading. Such a spontaneous back switching may go beyond the pulse time limit and then it will not contribute to SS with the negative sign but will contribute to OS loss [15]. Thus, one can propose that imprint contributes mostly to retention loss in the device in TiN TE, while the retention loss in the device with Ru TE is primarily affected by depolarization.

**Figure 6:**
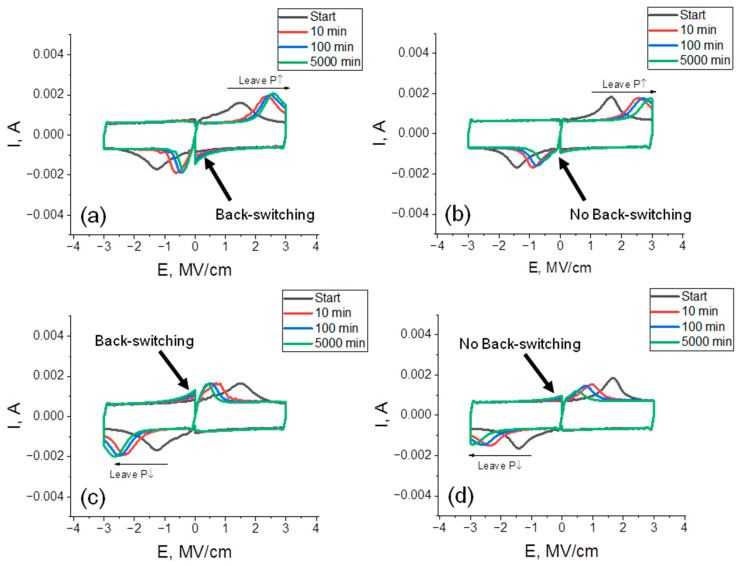
Dynamic I_sw_-E curves of the devices with TiN TE (**a**,**c**) and Ru TE (**b**,**d**) measured at the pristine state and after baking at 85 °C for 10, 100 and 5000 min.

**Figure 7:**
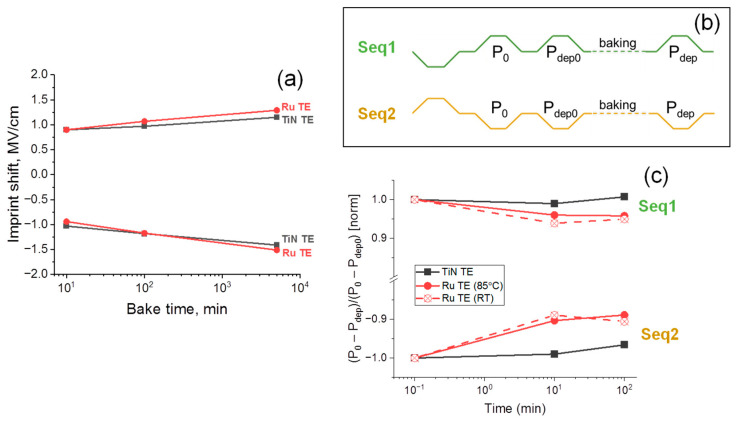
(**a**) Imprint shift estimated from the dynamic I_sw_-E curves; (**b**) the waveforms applied for measurements of depolarization-related loss of 2P_r_; (**c**) results of the depolarization-related loss of 2P_r_ measurements performed after different times of storage at 85 °C (solid lines) and at RT (dotted lines) from the device with TiN TE (black lines) and Ru TE (red lines). Lines serve as a guide for the eye.

To verify this assumption and to gain a deeper insight into the retention loss behavior, we performed dynamic I_sw_-E measurements before baking and after the same baking times as in retention measurements for both devices (Figure 6). The major difference between the dynamic I_sw_-E curves is that the valuable back-switching effect is seen when the device with TiN TE is measured after baking (Figure 6a,b), while there is almost no spontaneous back switching in the device with Ru TE (Figure 6c,d). Although this observation nicely confirms the assumption made above, the reason behind such a difference is not clear, especially taking into account rather high imprint shifts, which are observed in both devices. Figure 7a summarizes the imprint shift data. One can see that the imprint shifts are very similar. Moreover, it is even slightly higher in the device with Ru TE when the P↑ state is stored. From first sight, the considerably wider I_sw_ peaks and lower coercive field (E_c_) in the device with TiN TE handle such behavior. Indeed, it can be understood that when an I_sw_-E curve with wider I_sw_ peaks and a lower E_c_ is shifted, some fractions of I_sw_ peaks have more chances to cross the zero E axis, which will manifest itself in spontaneous back switching. However, the nature of such larger width is not clear. One can compare the width of the hysteresis measured after wake-up quasi-statically (Figure 2a,b). The hysteresis widths are almost identical, which means that the difference occurs at higher measurement frequencies. Considering the fact that both devices are rather similar in terms of RC values because they formed with identical TE and BE (except the very thin Ru inset, which has similar resistivity to TiN because of the low thickness) and the difference in *k*-values of HZO in both cases is not very high, we believe that the inherent polarization dynamics are different in two samples. One can notice that before baking, the I_sw_-E curve for the device with TiN TE is pinched more. Probably, the above-discussed t- to o-phase transition, more characteristic of this device, occurs with a time scale of the dynamic I_sw_-E curve measurements, resulting in such a pinching of the curve and a larger width.

Finally, we performed measurements that could better differentiate the depolarization-related retention loss from the other retention loss contributions. The pulse sequences for the measurements are presented in Figure 7b. The first pulse in each of the sequences puts the capacitor in the defined polarization state, upward (P↑) and downward (P↓) for the first (Seq 1) and second sequence (Seq 2), respectively. During the application of the second pulse with the opposite polarity, the 2P_r_ before baking is measured (designated as P_0_). During the application of the third one with the same polarity, the part of polarization, which could be lost in a short period between the second and third pulse (τ = 1 µs), is measured (designated as P_dep0_). Finally, the application of the fourth pulse with the same polarity after the different times of baking will allow for measuring the 2P_r_ lost because of the depolarization during the baking time (designated as P_dep_). In the result, the dependency of (P_0_ − P_dep_)/(P_0_ − P_dep0_) on the baking time, reflected by the part of the polarization that did not suffer from the depolarization, was received (Figure 7c). It should be noted that each of the measured values (P_0_, P_dep0_, P_dep_) also contain the contributions from leakages and the difference between the displacement currents at the rise and fall of the related voltage pulses. However, they cancel each other out after the mutual subtraction. One should also notice that the differentiation between imprint and depolarization by such an experiment is possible because the state written before the baking becomes even more stable during baking, while the depolarization, if it is, negatively affects the stability of the written state.

According to Figure 7c, there are no signs of depolarization in the device with the TiN TE, at least within the time scale of the experiment, while depolarization exists in the sample with Ru TE. Interestingly, we obtained similar results when baking at 85 °C was replaced with storage at RT. Such a similarity is the principle is not unexpected because the depolarization arises from the imperfect screening, which should not be so dependent on the storage temperature as the imprint. Thus, summarizing the retention-loss-related experiments, we conclude that imprint possibly intensifying by the involvement of the field-induced ferroelectricity (t- to o-phase transformation) is responsible for the retention loss in the device with TiN TE, while both depolarization and imprint contribute to the retention loss in the device with Ru. While the reasons behind such a difference are not clear, there is a temptation to connect both retention-related peculiarities with a difference in the crystal structures of HZO. While the involvement of the field-induced ferroelectricity (t- to o-phase transformation) is suggested to be because of the more preferred conditions for the t-phase formation and its continuous contention with the o-phase, the activation of the depolarization mechanism may be because of the non-ferroelectric m-phase in the bulk of HZO, which does not transform to o-phase and persists in the poled state or difference in the formed interfacial layers at the Ru/HZO and TiN/HZO interfaces in terms of thickness and/or k. Deeper investigations are required to identify this problem.

## 4. Conclusions

Overall, we can conclude that Ru TE shows no reliability improvement as compared to TiN, at least when one applies it to the HZO-based stacks in combination with automatic annealing at 400 °C, compatible with BEOL technology. There is only a valuable improvement in endurance, which can be linked with the lower scavenging ability of Ru as compared to TiN and consequently decreased V_O_ concentration; however, it is accompanied by huge fatigue, suggesting that Ru does not protect HZO against V_O_ generation during prolonged cycling, which most probably are deeper traps as compared to the traps formed at the interface with TiN TE. Although Ru slightly improves the OS retentions compared to TiN, SS (NSS) retention is actually worsened, which was connected to the contribution of the depolarization mechanisms with maintaining the high imprint shift with prolonged storage. The presented data reveal that the mechanisms behind the reliability issues in HZO-based capacitors are dependent on the electrode and the electrode-formation conditions and there is still a lack of a comprehensive understanding in the underlying phenomena that could predict the reliability properties in different stacks. To fill the existing understanding gaps, in situ investigations on the oxygen distributions in HZO by, for example, synchrotron photoelectron spectroscopy measurements, should be performed at each step of the reliability experiments. Moreover, the in situ diffraction or transmission electron microscopy measurements should accompany such investigations. Although such experiments are rather time and resource consuming, the strong interplay between the crystal structure and oxygen content, along with behavior features not specific to any particular mechanisms among the proposed, usually makes other indirect approaches highly speculative.

## Data Availability

Data are available at reasonable request from the authors.
